# Financial disclosure quality and sustainability disclosure quality. A case in China

**DOI:** 10.1371/journal.pone.0250884

**Published:** 2021-05-28

**Authors:** Indra Abeysekera, Feng Li, Yingjun Lu

**Affiliations:** 1 Discipline of Accounting and Finance, Charles Darwin University, Darwin, Australia; 2 Discipline of Accounting, University of Wollongong, Wollongong, Australia; 3 School of Accounting, Shanghai University of International Business and Economics, Shanghai, China; Universita degli Studi di Pisa, ITALY

## Abstract

This paper empirically examines whether there is an association between financial reporting disclosure quality and sustainability disclosure quality of the top 100 socially reputed Chinese listed firms. The paper computed financial disclosure quality by empirically combining earning qualities of accrual, persistence, predictability, and smoothness. Using content analysis and survey questionnaire research methods, it calculated sustainability quality by combining disclosure quantity (through quantitative weightings), disclosure type (through qualitative weightings), and disclosure item importance (through qualitative weightings) of economic, social, and environmental disclosures made in annual and sustainability reports, ascertained using the Global Reporting Initiative sustainability framework. The study finds that sustainability disclosure in the current period is sufficiently associated with financial disclosure quality of the current period and future period. Consistent with stakeholder theory, firms with a social reputation are perceived as trustworthy by stakeholders and shareholders. The findings lead to a cultural stakeholder theory where underlying values of societal culture create a condition supporting mutual stakeholder relationships between firm and various stakeholders. Demonstrating trustworthiness through disclosures can help boost consumer confidence and foreign trade relations for Chinese firms. The Chinese government can design innovative schemes to reward and promote trustworthiness in firms, such as regulating base-point reductions in interest rates on borrowing or raising funds.

## Introduction

Corporate history cites several stories of financially sound firms that managed their social and environmental activities by being less responsive to stakeholders, culminating in social, political, and social costs to them and to society. Such events also erode the trust relationship between businesses and stakeholders. The political, social, and economic environment in a country can influence the nature of such relationships. A way to build trustworthiness is through disclosure, but it is not the transparency of disclosures that is paramount but their integrity, to build trustworthy relationships.

From a political perspective, China follows a regulatory approach with a high degree of authoritarianism where rule of law is selectively applied [1]. From an economic perspective, it has built a strong manufacturing base to export, and has a growing economy [2]. From a social perspective, Confucianism is a foundation of the societal culture, which values relationships built upon on trustworthiness, and considers them a virtue [3]. These perspectives make China unique for this study, which aims to examine unregulated disclosures as a vehicle to build trustworthy relationships between firms and stakeholders.

China hosts a dense population despite its large land base, and the increasing economic activity has seen the country emerge as a world economic powerhouse. The growing economic opportunities for Chinese firms to manufacture and trade domestically and overseas have given the capacity for opportunistic managerial behaviour that may undermine firms’ responsible conduct towards the society and the environment. Environmental contamination from chemicals and exudates from landfill sites that pollute the earth, water, and air has to led to public protests by Chinese citizens [4]. A nationwide pollution survey conducted in 2019 confirmed unacceptable levels of air and water pollution in China, although with a progressive decrease over the past decade [5, 6]. Although the COVID-19 crisis decreased air pollution due to diminished manufacturing activities, it has now returned to the pre-COVID level in China [7]. The result is not only environmental issues, but also challenging economic and social issues to address: public emergencies and perceived health threats are public concerns [8].

Socially reputable firms have made an image for sharing business information relevant to stakeholders [9]. In a corporate context, the two major groups are shareholders as one stakeholder group, and other stakeholders as the second group. Socially reputable firms have earned the trust of stakeholders. Stakeholders’ trust means they willingly become vulnerable [10]. Firms that stakeholders do not perceive as trustworthy can erode their trusting relationship.

Disclosures are a cornerstone of transparency that can decrease corruption and mismanagement. Disclosures communicate information to various stakeholders who have access to and are willing to receive such information [11]. Firms make financial disclosures to shareholders and these disclosures are also important other stakeholders. Firms make non-financial disclosures to other stakeholders, and these are also important to shareholders. What is important is whether these disclosures are relevant to the stakeholders, and increase the quality of information shared with them. Sharing high-quality information with stakeholders can increase the trustworthiness of businesses; otherwise, stakeholders can become vulnerable in that relationship with the firm [11]. The trustworthiness earned by companies can translate into product sales, lower cost of capital, and so forth, resulting in benefits to firms.

Although research has examined the relationship between disclosures and performance of reputable firms, the relationship in firms making disclosures to stakeholders has received limited attention. The relationship between stakeholder-relevant information and financial information–what should be reported based on stakeholders’ expectations–is sparse, especially concerning the quality of disclosures. Further, such examination is mostly absent in the context of China. China is politically regulated, which filters into the business landscape, has rapid economic growth with a strong manufacturing base, and is culturally founded on Confucianism, all of which makes China a unique and vital research site.

Five aspects bring uniqueness to this study. First, previous studies have used social or environmental dimensions, or have considered both, for corporate social responsibility (CSR) performance and disclosure. They have primarily omitted the economic aspect. The Global Reporting Initiative (GRI) sustainability framework adopted in this paper includes economic, social, and environmental aspects as interconnected dimensions, a point of difference from previous studies. Second, previous studies have examined social and environmental aspects from the researcher or database collector’s perspective. This paper collects and analyses corporate sustainability information relevant to stakeholders. Third, previous studies have singled out discretionary accruals as a proxy for earnings quality. This paper considers financial reporting quality, comprising four earnings qualities that drive sound financial statement reporting. Fourth, China has a unique context, as a fast-growing economy founded on Confucianism. Fifth, the literature has examined social reputation as a context with CSR performance [9, 12]. Still, there is little empirical evidence about the association of stakeholder-relevant sustainability disclosure of firms with financial reporting quality.

In this paper, we selected the top 100 socially reputable firms in China. We argued that financial disclosures made relevant to shareholders (financial disclosure quality) are associated with economic, social, and environmental disclosures having relevance also to other stakeholders (sustainability disclosure quality). In this way, we ascertained that firms maintain trustworthiness across all stakeholders.

The regression analysis conducted shows that sustainability disclosure quality is associated with financial disclosure quality. Sustainability disclosure quality of the current year is closely associated with the financial disclosure quality of the current year and the future year. The next section discusses the relevant literature to show the need to conduct this examination. The theory section outlines the theoretical basis for the study and research hypotheses. After that, the methodology section describes selection of the sample and data sources. The results section presents the empirical results. The last section provides concluding remarks with possible future research propositions.

## Relevant literature

### Corporate sustainability

Corporate sustainability originated from the World Commission on Environment and Development definition of meeting needs without compromising the needs of future generations [13]. Since then, sustainability has branched out with an ecological emphasis, considering a triple bottom line. The triple bottom line has a financial reporting emphasis complemented by social and environmental reporting. Sustainability disclosure is where social and environmental aspects are given the same level of importance as financial disclosures [14]. The focus of sustainability is the peaceful co-existence of the environment with economic development in which society plays a vital role [15]. Corporate sustainability research has used first-hand data collected from surveys and interviews, as well as analysing annual reports [16]. This paper uses the term sustainability rather than CSR for the following two reasons. First, it examines both social and environmental aspects, taking the view that these are interconnected with economics, and it measures disclosures using annual reports and collecting first-hand data. Second, the study uses the GRI [17] sustainability framework to collect data about the economy, society, and environment.

Research points out that firms create value primarily to benefit shareholders. Firms either retain the created value to expand their activities in the future or immediately distribute it to the shareholders. The institutionalising of the economic, social, and moral environments supports a consumptive society that exercises individual will and promotes consumption. We can derive the meaning of this societal behaviour from Hofstede [18], where certain countries score high on individualism (i.e., looking after oneself and immediate family only) and indulgence (i.e., willingness to realise impulses and desires to enjoy life and have fun). Hofstede individualism and indulgence scores are high for Australia (90, 79), Canada (80, 68), New Zealand (79, 75), the United Kingdom (89, 69), and the United States (91, 68). These cultural settings are conducive to sharing firms’ created value based on contracts and transactions; in these societal cultures, shareholders are an acceptable fit as the primary beneficiary of firms’ value creation.

### Sustainability disclosure quality

Firm behaviour must mimic societal behaviour to become acceptable by societal cultures that build relationships on contracts. The literature points out that in Western settings, firms use disclosures. There are various theoretical posits used to explain organisational sustainability disclosure response to stakeholders. Firms make disclosures about past and present events to meet societal contracts. They manage their impressions by viewing stakeholders as having a coherent, unified set of norms (i.e., legitimacy theory), and make incremental voluntary disclosures about past and present events (i.e., signalling theory). In their sustainability disclosure, firms can go beyond meeting societal needs for legitimacy and adopt hypocrisy, by giving false assurances that they will realise reporting about future events (i.e., organised hypocrisy theory). Further, acknowledging that there are different stakeholder groups with varying and even conflicting interests, firms use a range of rational disguises in disclosures to appease various stakeholders (i.e., organisational façade theory) [14, 19]. Because firms manage stakeholder relationships with rational morality in the marketplace, some researchers suggest that there must be regulatory intervention mandating sustainability disclosure for the public interest (i.e., regulatory capture or economic theory of regulation) [14]. The 2014 European Commission directives require certain issuers to provide specific sustainability disclosures starting in 2018 [20]. The investing community has made repeated requests to the Securities Exchange Commission to mandate sustainability disclosure to show firms’ economic effectiveness. One proposal from a researcher is to include a section called Principle-Based Sustainability Disclosure and Analysis that addresses at least three of the most crucial sustainability issues. The directors certify the accuracy of disclosure for their explained impact on firm performance [21]; accuracy extends a direct association between the two. Research points out that in several Western country settings, mandatory regulatory powers of governments can positively influence firm environmental strategies and performance [22]. In the EU setting, an EU directive required large companies to mandatorily disclose non-financial information. A study that examined non-financial disclosure relating to society, environment, and governance found that larger firms, firms in sensitive industry sectors, firms having larger board size, and firms with more shareholders made more disclosures [23]. A study conducted with Bangladeshi Banks showed that regulatory pressures can increase authentic sustainability disclosure [24]. Research conducted in Peru found that regulatory pressures had no impact on sustainability disclosure quality, an index constructed by researchers. The Peruvian study pointed out that one reason for such difference may arise because some countries follow the regulatory intervention of firms to disclose and explain, whereas other countries (for example, India) follow firms to disclose or otherwise be penalised [25]. These studies show that the impact of regulatory intervention and sustainability disclosure quality are unresolved research agendas requiring clarity through further research.

A research finding with firms in the USA suggests that sustainability disclosure quality (primarily devised by binary variables) can associate with higher innate earnings quality (earnings fluctuations inherently associated with the firm such as firm size, operating cycle, cash flows volatility, sales volatility, negative earnings, leverage, and capital intensity) and discretionary earnings quality (earnings fluctuations associated with management discretionary behaviour), contesting the applicability of organisational hypocrisy theory and organisational façade theory [12].

These works point out that in Western societal settings: (1) firm sustainability disclosure aims to increase useful information to shareholders; (2) firms can manage sustainability disclosures to various stakeholders and may not necessarily be truthful about the future; and (3) this societal setting requires regulatory intervention to improve the performance of sustainability disclosures. China is low in individualism score (20), meaning that social actors (firms, individuals) must look after each other. The indulgence score is also low (24), meaning that people restrain their enjoyment and consume with restraint [18]. These contrasting Hofstede scores on cultural dimensions point to China adopting an organically developed relationship-focused stakeholder orientation built upon trustworthiness, where performance and disclosures are likely to match closely, thereby taking care of different stakeholders’ needs [26].

### Financial reporting quality

Although earnings reported are regulated by International Financial Reporting Standards (IFRS), and Chinese Accounting Standards are considered equivalent to IFRS [2], their computation is subject to some level of discretion on the part of managers, which can lead to managing earnings. It is not easy to estimate earnings management because it refers to an intentional change of financials to ’harm’, over the natural variation [27]. The real activity of earnings management changes earnings to change cash flows. Accruals earnings management changes estimates and accounting policies. Although earnings management can lead to low-quality earnings, the lack of earnings management does not necessarily increase earnings quality. High-quality earnings are conservative–the earnings must sustain over time, and be unbiased. Using a survey conducted with Chief Financial Officers inquiring into earnings quality, Nelson and Skinner [28] found that sustainable and predictable earnings are of high quality. Rather than flipping accruals quality (an earnings management quality) for earnings quality, our study focuses on four earnings qualities in acknowledgement of multiplicity. The chosen earnings qualities appeal to conservative earnings, leading to more useful financial statements, which are helpful to stakeholders contracting with the firm.

### Reputation as a context

Research shows that reputation helps firms to build trustworthiness with stakeholders [29–31]. Organisational reputation comprises a set of financial and non-financial attributes [32], and is about stakeholders’ perception that the firm sincerely cares about their interests [33, 34]. Trustworthiness is a unique asset that cannot be copied, imitated, or bought as a single asset. Organisational reputation is a reliable indicator of genuinely trustworthy behaviour because it does not fade over time [35]. Research shows that financial reporting quality is higher in socially reputable firms than those that are not, because reputable firms have a public image to safeguard [36]. They are less likely to engage in earnings management, and report higher quality earnings [12]. Research that examined 57 firms listed on the Dow Jones Sustainability Index in 2003 (matched by size, country, and industry) showed a positive association between reputable firms and sustainability disclosure quantity [9]. The literature, however, has not examined the socially reputable context in China, with sustainability disclosure that stakeholders care about as well as financial reporting quality comprising four earnings quality measures.

## Theoretical framework and hypothesis development

Legitimacy theory takes the view that a firm has a compelling need to meet the social demand in order to exist, with an intent to advance its economic interest. In the context of socially reputable firms, there is no compulsion for them to meet social demands because they are already enjoying the status of reputation offered by society. The socially prominent firms are driven by doing the right thing for stakeholders. Rather than theorising as logically calculated activities, this paper examines firms’ behaviourally responsive actions undertaken to build relationships.

Further to the analysis and discussion in the preceding section on the relevant literature, stakeholder theory proposes that all stakeholders have intrinsic value, and therefore firms should attend to the interests of all stakeholders. Stakeholder theory has not made simplified assumptions of self-interest and hence opportunistic behaviours, instead emphasising an attitudinal and behavioural approach towards stakeholders. The relationship is about reciprocal trust between the firm and its stakeholders [37], with stakeholders expecting trustworthiness from firms engaging in ethically justifiable behaviour [38].

**Fig 1:**
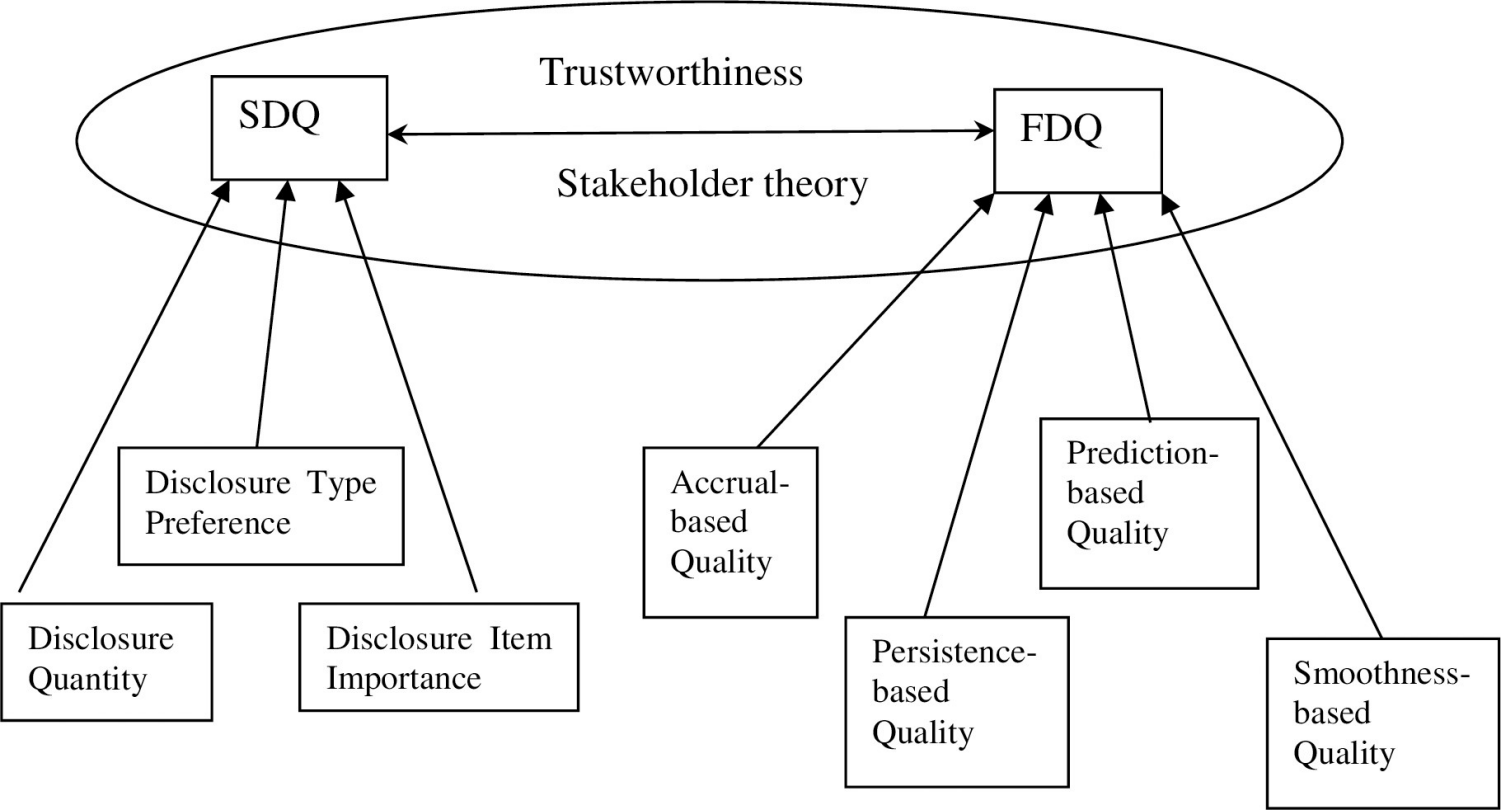
Theoretical framework.

Freeman and Phillips [39] make us understand stakeholder theory as a way of conceiving strategy and ethics as essential attributes in managing relationships with stakeholders, as directed by managers. They do however point out that stakeholder theory is not one but a genre of theories comprising at least a liberal perspective and a socialist perspective. In this paper, we examine the stakeholder theory applicable to China grounded on trustworthy relationships that are mutually shared and reciprocated, as shown in Fig 1.

The interconnected stakeholders in the contemporary information age form a single ecosystem. When firms prioritise one stakeholder group over another, such information is more commonly shared in the ecosystem and can erode the trust of the affected stakeholder group [40]. Socially reputed firms are more likely to ensure that their reputation is maintained or even enhanced in the stakeholder ecosystem. In the ecosystem that shares information, we expect firms making stakeholder-relevant sustainability disclosures to produce higher quality financial disclosure to shareholders to maintain and enhance their trustworthiness. Building trustworthy relationships with stakeholders is about upholding integrity rather than transparency. Because in trusting firms’ behaviours, stakeholders have accepted to become vulnerable, firms have a moral duty to meet their expectations by making relevant and reliable disclosures.

China is on the top of the list of Hofstede’s [18] long-term orientation of societal culture dimension scores where relationships underpin score construction. Chinese society considers relationships as a vital part of conducting affairs, embedded in their values, norms, and cultural system [26]. Chinese capitalism is embedded in this cultural rooting, making it distinct from other forms of capitalism. A core concept in the Chinese relationship building is guanxi, a word with no direct English translation. Guanxi can be understood through its direct connection to two other Confucian virtues: xinyong–trustworthiness, and renqing–mutual obligations [41]. A long-term orientation score indicates that the Chinese build trust over a period of time, not because they lack rules and laws imposed through their legislative mechanisms, but because trustworthy relationships are a way of building connections. Emphasis on mutual obligation over trustworthiness may lead to exchanging gifts and favours, as well as corrupt practices. Reputable firms, on the other hand, have a focus on trusting relationships over mutual obligation. A way to bring meaning to guanxi with the two interrelated Confucian virtues is that the relationship is underpinned by trustworthiness and supported with mutual commitments; it is a social tool for business operations [42].

This study, therefore, expects that the current year Sustainability Disclosure Quality (SDQ_t_) associates with the current year financial disclosure quality (FDQ_t_). The credible quality signal in the information ecosystem becomes a trusting relationship between socially reputable firms and not only shareholders (i.e., financial stakeholders) but also other (i.e., non-financial) stakeholders.

Considering the above discussion, we present the following hypotheses for the empirical testing of firms in China, and state them without a direction because we cannot conclude which one influences the other, as all stakeholders share firms’ trustworthiness. Sharing trustworthiness in a shared ecosystem applies to China because of the cultural emphasis on mutually shared trustworthy relationships. We state our first hypothesis as follows.

H1: Sustainability disclosure quality (SDQ_t_) in the current period associates with financial disclosure quality (FDQ_t_) in the current period.

The building of trusting relationships is an ethical core [31], and in China (which according to Hofstede has the highest long-term orientation score) requires a certain length of attachment and develops over time; and, it feeds on reputation [33, 43]. Sustained trustworthiness has a more pervasive effect over the period. For example, firms aspiring to become trustworthy with high-quality sustainability disclosures made in this period can associate with the trustworthiness of high-quality financial disclosure not only in the current period but also in the future period.

We expect that SDQ_t_ in the current period also associates with FDQ_t+1_ in the future financial year, and state the second hypothesis as follows.

H2: Sustainability disclosure quality (SDQ_t_) in the current period associates with financial disclosure quality (FDQ_t+1_) in the future period.

## Research methodology

### Sample

This paper examined 100 firms with social reputation, as ranked in a list initiated and published in a very popular newspaper in China, Southern Weekend. This is the same sample of firms as used by Lu and Abeysekera [44, 45]. The 2008 Chinese Social Responsibility List of stock-listed firms is compiled using specific criteria developed by a group of experts and scholars from government, industry, universities, and research institutes. Firm selection is mediated by trade unions (Federation of Trade Unions), chambers of commerce (All-China Federation of Industry and Commerce), and reputable universities (Peking University, Fudan University, and Nankai University).

A study conducted with Bangladeshi banks measured sustainability disclosure quality using 11 items constructed by the researchers, comprising 5 items about relevant information and 6 items about reliable information [24]. A study conducted in Peru ascertained sustainability disclosure quality using 23 items constructed by the researchers. These items comprise 9 credibility-related items, 11 content-related items, and 3 communication-related items [25]. This study differs from previous studies in measuring sustainability disclosure quality in five ways. First, it uses the GRI framework for item measurement. Second, it measures disclosure quantity. Third, the study measures disclosure quality in two dimensions–disclosure type preference, and disclosure item importance. Fourth, in measuring disclosure quality, the study consulted relevant stakeholders about their preference for disclosure types and importance of GRI items. This approach appreciates that stakeholders are distinguishable in sustainability disclosure, and it is more precise in that firms can specifically discharge accounting for sustainability to different stakeholders [46]. Fifth, the sustainability disclosure quality of this study is the combined effect of disclosure quantity, stakeholder-relevant disclosure type preference, and stakeholder-relevant disclosure item importance. The three dimensions of sustainability disclosure quality are presented below.

### Disclosure quantity

The study measured sustainability disclosure quality using the methodology presented by Lu and Abeysekera [44, 45] where the 121-item GRI sustainability framework is used to collect data. The study examined the content of annual reports and CSR reports of firms for the frequency of GRI item disclosure by conducting latent content analysis in search of the meanings defined for the GRI items in the GRI framework. The study examined both annual reports and CSR reports of firms, where applicable, because stakeholders are likely to obtain information from both reports. Another researcher independently examined a sample of 25 firms’ reports. The comparison of scores showed an overall agreement (Krippendorff’s alpha) of over 95 per cent.

### Disclosure type preference

The methodology asked stakeholders about their disclosure presentation preferences: (1) general story type; (2) specific story type; (3) presented as numbers; (4) displayed as numbers with benchmarking for comparison; or (5) presented as numbers with details on its makeup. Because stakeholder groups differ in their interests, this study designed six versions of the questionnaire for the six identified stakeholder groups: economic, environmental, labour, human rights, societal, and product. Each questionnaire contained some GRI items that were common to all questionnaires because they are relevant to all stakeholder groups. The remainder of the questions were about GRI items relevant to each stakeholder group, i.e., they were different for each questionnaire. There is no public registry of stakeholders as found and maintained with shareholders in firms. Consequently, the study emailed the six versions of the survey questionnaire to the 100 firms, to the attention of the executives who take charge of CSR and annual reports. These executives were requested to distribute the questionnaires to relevant stakeholders and subsequently collect them.

In the survey questionnaires, respondents assigned a preference score between 0 and 100 to each of the GRI items based on perceived importance, thereby stating their preference for the type of disclosure.

### Disclosure item importance

Because different stakeholder groups pay attention to GRI items that are relevant to them, the study also consulted 12 stakeholders representing diverse stakeholder groups under the GRI framework. These were: (1) a large individual shareholder, (2) a manager of an institutional shareholder, (3) a banking loan manager, (4) a chief officer of a government authority, (5) an academic, (6) an auditor partner, (7) a human resource manager of the firm, (8) an employee representative, (9) a customer representative, (10) a manager of a major supplier, (11) a representative of the local community, and (12) a local media manager. The study asked them to rank each of the 121 items in the GRI framework (i.e., 42 context items and 79 performance items) according to how important it is to them, scoring 0 to 4.

The methodology computed Sustainability Disclosure Quality (SDQ) by combining disclosure quantity, disclosure type, and disclosure item importance, as follows:

$$SDQ\;score=\sum_{j}{}^{0\;to\;\infty }\;*\sum_{j}{}^{1\;to\;100}\;*\sum_{j}{}^{0\;to\;121},\;j=firm$$

### Financial disclosure quality

The use of Chinese Accounting Standards and IFRS by Chinese listed firms can give rise to variations in the estimates of accruals, estimating the smoothing of earnings, and their influence on computing persistent and predictable earnings. These estimations can undermine the quality of earnings data. To capture an array of qualitative aspects, financial disclosure quality includes four earnings qualities: accrual-based earnings quality, persistence-based earnings quality, predictability-based earnings quality, and smoothness-based earnings quality. The earnings data for the four earnings qualities were obtained from the Chinese Stock Market Analysis Research database to measure the four earnings qualities.

The literature proposes different ways to measure accruals quality. This paper used the model that attempts to map total current accruals as the outcome variable, with past, present, and future cash flows as independent variables, using total assets to standardise the mapping across firms [36, 47–49]. A lower standard deviation of the residual indicates higher accruals quality (AQ_j,t_), a dimension of financial reporting quality.

The accruals quality equation is as follows. All variables are scaled by the beginning total assets.

TCA _j, t_ = a + b_1_* CFO _j, t-1_ + b_2_* CFO _j, t_ + CFO _j, t+1_ + c

Where:

TCA _j, t_      Firm j’s total current accruals in t (ΔCA _j, t_− ΔCL _j, t_− ΔCash _j, t_ + ΔSTDEBT _j, t_ + Δ TP _j, t_);

Total Asset _j, t−1_ Firm j’s total assets in year t-1;

CFO _j, t-1_        Firm j’s cash flow from operations in year t-1;

CFO _j, t_      Firm j’s cash flow from operations in year t; and

CFO _j, t+1_        Firm j’s cash flow from operations in year t+1.

The model used earnings to measure the persistence dimension of financial reporting quality, by mapping current earnings as an outcome variable with past earnings as an independent variable, standardised by total assets across firms. The strength of the past earnings with current earnings, identified with the coefficient parameter, indicated earnings persistence (PER_j,t_), with 1 being the perfect persistence [36, 48–50]. The persistence equation is as follows. All variables are scaled by the beginning total assets.

Earn_j,t_ = a + b_1_* Earn_j,t-1_ + c

Where:

Earn _j, t_ Firm j’s net income before extraordinary items in year t;

Earn _j, t−1_ Firm j’s net income before extraordinary items in year t-1, and

c Residual.

In the persistence regression, the variance in the residual indicates earnings predictability [49, 50]. More substantial variations in the residual indicate lower persistence, measured as the square root of that variance.

PRED_j,t_ = square root (б2 (cˆ _j,t_)

Where:

PRED_j,t_ Earnings predictability of firm j in year t; and

σ2 (cˆ _j, t_) Estimated-residual variance of firm j in year t, calculated from Equation

The mapping of earnings as denominator with cash flows as nominator, both standardised by total assets across firms, as a ratio, shows the earnings smoothness to accomplish the desired cash flow position. Higher values indicate more earnings smoothness, and lower values mean less. The smoothness equation is as follows. All variables are scaled by the beginning total assets [48–50].

Smoothness = CFO _j, t_ / Earn _j, t+1_

Where:

σ      Firm j’s standard deviation;

CFO j,t Firm j’s operating cash flows in year t (indirect approach); and

Σ (Earn j,t) Firm j’s net income before extraordinary items in year t.

In this paper, financial reporting quality comprises four earnings qualities. We ranked each earnings quality item on a scale from 1 to 10, with 1 being the lowest and 10 being the highest. In accruals quality, predictability, and smoothness, higher values indicate higher earnings quality and are ranked on an ascending scale. Higher persistence earnings show lower earnings quality and are listed on a descending scale. There is no one accepted way to combine the four earnings qualities, and this study used the standardised average of the aggregate score of the four ranked dimensions to measure the FDQ [51].

FDQ_i_ = [AQ_j_^1 to 10^ + PER_j_^1 to 10^ + PRED_j_^1 to 10^ + SMOOTH_j_^1 to 10^] /4

### Control variables

The literature has identified several variables relating to sustainability disclosure and financial reporting disclosure. The model included these variables: firm size, cash flows from operations in the current year, annual sales, length of the operating cycle, earnings losses reported in past years, debt level, and (non-current) capital intensity, which are known to influence this empirically tested relationship with innate earnings quality [12, 52–55].

Table 1 describes the control variables.

**Table 1:** Control variables.

Variable abbreviation	Meaning
SIZE	Natural log of total assets (in millions of dollars)
CFO	Calculated using a rolling average of five years of cash flows from operations adjusted with total assets at the beginning of the period
SALES	The standard deviation of sales revenue scaled by beginning total assets (both in millions of dollars), computed using a five-year rolling window ending in the current year
LEVERAGE	Total assets over short- and long-term debt
CAP INTENSITY	The ratio of the net book value of property, plant, and equipment to total assets
OP CYCLE	Natural log of the sum of days of accounts receivable and days of inventory: [(360/ (sales revenue/average accounts receivable) +360/(cost of goods sold/average inventory) (all in millions of dollars)]
NEG EARNINGS	The frequency of negative net income before extraordinary items over five prior years
t	Current year
j	Firm

The two main regression models that regressed FDQ_t_ on SDQ_t_ to test the hypotheses are stated as follows.

H1 test statistical model:

FDQ_t_ = α_0_ + β_1_ SDQ_jt_ + c_1_ SIZE_jt_ + c_2_ CFO_jt_ + c_3_ SALES_jt_ + c_4_ OP CYCLE_jt_ + c_5_ NEG EARNINGS_jt_ + c_6_ LEVERAGE_jt_ + c_7_ CAP INTENSITY_jt_ + c_8_ FDQ_t-1_ +є_jt_

H2 test statistical model:

FDQ_t+1_ = α_0_ + β_1_ SDQ_jt_ + c_1_ SIZE_jt_ + c_2_ CFO_jt_ + c_3_ SALES_jt_ + c_4_ OP CYCLE_jt_ + c_5_ NEG EARNINGS_jt_ + c_6_ LEVERAGE_jt_ + c_7_ CAP INTENSITY_jt_ + c_8_ FDQ_t_ + є_jt_

Where:

FDQ is the standardised average of the ranked four earnings qualities (accruals-based, persistence-based, predictability-based, and smoothness-based); and SDQ_t_ is the product of social and environmental reporting quantity* stakeholder relevance by information type * stakeholder relevance by item importance.

## Results

### Descriptive statistics

Table 2, which describes sample statistics, shows that FDQ_t_ and FDQ_t+1_ are similar and follow a normal distribution. SDQ_t_ also follows a normal distribution. They were similar sized firms. Firms differed widely in operating cash flows, sales, and leverage; fewer firms showed high cash from operations, high sales, and high leverage. The firm size and the operating cycle follow a normal distribution. Most firms were asset-rich, and only a few firms had experienced earning losses in the past five years. Most firms had a low density of non-current assets compared to their total asset base.

**Table 2:** Descriptive statistics.

Variable	Mean	Std. deviation	Median	Minimum	Maximum
FDQ_t_	22.05	7.94	21.5	7	39
FDQ_t+1_	21.93	8.56	22	5	37
SDQ_t_	201.28	82.12	189	82	522
SIZE	10.68	0.75	10.48	9.32	12.99
CFO	83.66	203.78	11.04	0.76	1324.15
SALES	160.78	389.71	63.33	7.12	3191.93
OP CYCLE	1.76	0.33	1.75	0.90	3.10
NEG EARNINGS	0.18	0.58	0.00	0	3
LEVERAGE	3.48	5.32	1.67	0.22	30.40
CAP INTENSITY	0.28	0.19	0.29	.01	0.69
FDQ_t-1_	21.88	7.37	22.0	8	37

Table 3 indicates a positive association of SDQ_t_ with FDQ_t_ and FDQ_t+1_. Control variables (except operating cycle, negative earnings, and capital intensity) are not significantly associated with FDQ_t_. Lower operating cycle firms are likely to have a closer match between operating cash flows and accruals. Firms in more capital industries with higher non-current tangible assets are likely to contribute to a greater mismatch between operating cash flows and accruals.

**Table 3:** Correlation matrix of the FDQ_t_ and FDQ_t+1_ of the main model.

Variable	FDQ_t+1_	FDQ_t_	SDQ	SIZE	CFO	SALES	OP. CYCLE	NEG. EAR	LEV	CAP. INT	FDQ_t-1_
FDQ_t+1_	1										
FDQ_t_	0.565***	1									
Probability	0.000										
SDQ_t_	0.535***	0.493***	1								
Probability	0.000	0.000									
SIZE	0.458***	0.498***	0.665***	1							
Probability	0.000	0.000	0.000								
CFO	0.353***	0.380***	0.591***	0.681***	1						
Probability	0.000	0.000	0.000	0.000							
SALES	0.306***	0.286***	0.512***	0.415***	0.319***	1					
Probability	0.002	0.004	0.000	0.000	0.001						
OP. CYCLE	-0.069	-0.215**	0.009	-0.018	-0.034	-0.079	1				
Probability	0.497	0.032	0.926	0.861	0.741	0.433					
NEG.EARN	-0.004	-0.020	-0.056	-0.001	-0.048	-0.073	-0.138	1			
Probability	0.972	0.846	0.580	0.995	0.635	0.474	0.170				
LEVERAGE	0.251**	0.243**	0.280***	0.555***	0.465***	-0.033	-0.059	0.319***	1		
Probability	0.012	0.015	0.005	0.000	0.000	0.743	0.558	0.001			
CAP.INTENSITY	-0.160	-0.160	-0.031	-0.238**	-0.357***	0.067	-0.088	-0.048	-0.489***	1	
Probability	0.112	0.113	0.758	0.017	0.000	0.508	0.383	0.638	0.000	
FDQ_t-1_	0.428***	0.417***	0.393***	0.269***	0.291***	0.331***	-0.064	-0.133	0.069	0.008	1
Pr	0.000	0.000	0.000	0.007	0.003	0.001	0.526	0.187	0.498	0.939	

***, **, *** denote significance at the 1%, 5%, and 10% levels, respectively. Probability refers to the statistical significance (alpha) value.

There is no high correlation among variables. The mean-variance inflation factor (VIF) for regression on the Hypothesis 1 testing model is 1.86 (maximum 3.03 and minimum 1.06), and the mean VIF for regression on the Hypothesis 2 testing model is 1.94 (maximum 3.2 and minimum 1.14), indicating no multiple collinear relationships.

Table 4 summarises the test results. The F value is statistically significant at one per cent for both models, indicating that the overall regression model fits the sample data and the model is statistically significant. This means the independent and control variables improve the model fit. The lagged FDQ_t-1_ positively associates with FDQ_t_ of the current period. SDQ_t_ is significantly associated with the financial disclosure quality of the current and future years. The model’s explanatory power has increased slightly from the current period (41.3 per cent) to the next period (41.9 per cent) FDQ.

**Table 4:** Relationship between financial disclosure quality and sustainability disclosure quality.

	Hypothesis 1 (FDQ_t_)			Hypothesis 2 (FDQ_t+1_)		
Variable	Coef.	Std. error	Pr	Coef.	Std. error	Pr
SDQt	0.026**	0.011	0.014	0.035**	0.014	0.012
SIZE	3.742***	1.332	0.006	0.543	1.929	0.779
CFO	-0.004	0.004	0.354	-0.003	0.005	0.510
SALES	0.001	0.001	0.186	0.001	0.001	0.462
OP. CYCLE	-5.277***	1.596	0.001	0.124	2.115	0.953
NEG.EARNINGS	0.231	1.111	0.836	0.128	1.054	0.904
LEVERAGE	-0.160	0.129	0.219	0.047	0.184	0.798
CAP.INTENSITY	-7.057*	3.874	0.072	-4.227	5.369	0.433
FDQ_t-1_	0.280**	0.108	0.011			
FDQ_t_				0.403***	0.112	0.001
Constant	-17.030	14.391	0.24	1.175	18.735	0.950
R2	41.3%			41.9%		
F	0.000			0.000		
Observations	100			100		

*** and ** denote significance at the 1% and 5% levels, respectively.

These results support both Hypotheses 1 and 2, with statistical associations between SDQ_t_ and FDQ_t_ and SDQ_t_ and FDQ_t+1_. As argued in presenting the case for this study, stakeholders trust firms for being credible and ethical, and these socially reputable firms ensure trustworthy relationships not only with shareholders but also with other stakeholders. The association between SDQ_t_ and FDQ_t_ show that firm size, operating cycle, and capital intensity (weak) as innate earnings qualities has a statistical significance in the current year. The SDQ_t_ and FDQ_t+1_ show that SDQ_t_ improves in statistical significance, complementing the influence of those innate earnings quality; stakeholders trustworthiness developed by firms through SDQ_t_ sustains into the future year with FDQ_t+1_.

### Robustness tests

#### Alternative multidimensional financial disclosure quality

The study used the measure suggested by Chen et al. [51] as an alternative measure for ascertaining financial disclosure quality, here known as FDQ(C), by computing the three earnings qualities: current accrual-based earnings quality, total accrual-based earnings quality, and discretionary revenue-based earnings quality (Table 5).

**Table 5:** Relationship between financial disclosure quality (computed as per Chen et al. [51]) and sustainability disclosure quality.

	Hypothesis 1 (FDQ(C)_(t)_			Hypothesis 2 (FDQ(C)_(t+1)_)		
Variable	Coef.	Std. error	Pr	Coef.	Std. error	Pr
SDQ	0.011***	0.003	0.000	0.007***	0.002	0.003
SIZE	1.105***	0.409	0.008	0.211	0.320	0.512
CFO	-0.002**	0.001	0.027	0.000	0.001	0.695
SALES	0.000	0.000	0.353	0.000	0.000	0.969
OP. CYCLE	-0.996**	0.440	0.026	-0.416	0.375	0.270
NEG.EARNINGS	-0.156	0.296	0.600	0.078	0.234	0.741
LEVERAGE	-0.038	0.042	0.366	-0.022	0.043	0.601
CAP.INTENSITY	0.011***	0.003	0.000	-1.116	0.881	0.209
FDQ(C)_t-1_	1.105***	0.409	0.008			
FDQ(C)_t_				0.609***	0.078	0.000
Constant	-7.992	4.172	0.059	-0.346	3.206	0.914
R2	44.3%			66.3%		
F	0.000			0.000		
Observations	100			100		

*** is at 1% level, and ** is at 5% level significance level.

The earnings quality values were decile-ranked and then aggregated. The F value is statistically significant at one per cent for both models indicating that the overall regression model fits the sample data and the model is statistically significant. This means the independent and control variables improve the model fit. As shown in Table 5, the study found a significant and positive association between the SDQ and the financial disclosure quality in the current year FDQ(C)_t_, and in the future year FDQ(C)_t+1_.

Table 6 shows the correlation matrix using the financial disclosure quality method of Chen et al. [51]. The VIF is 1.99, with a minimum value of 1.38 and a maximum of 2.48 for individual variables. This study assigns the inconsistencies in the behaviours of the control variables between the Chen et al. FDQ(C) model and the FDQ in the primary model, to the differences in measurements between the two financial disclosure quality measures.

**Table 6:** Correlation matrix of the FDQ of Chen et al. [51].

Variable	FDQ(C)_t+1_	FDQ(C)_t_	SDQ	SIZE	CFO	SALES	OP. CYCLE	NEG.EAR	LEV	CAP.INT	FDQ_t-1_
FDQ (C)_t+1_	1										
FDQ (C)_t_	0.780***	1									
Probability	0.000										
SDQ	0.610***	0.562***	1								
Probability	0.000	0.000									
SIZE	0.524***	0.508***	0664***	1							
Probability	0.000	0.000	0.000								
CFO	0.384***	0.332***	0.592***	0.681***	1						
Probability	0.001	0.007	0.000	0.000							
SALES	0.351***	0.334***	0.512***	0.4151***	0.319***	1					
Probability	0.003	0.007	0.000	0.001	0.001						
OP. CYCLE	-0.145	-0.153	0.0094	-0.018	-0.034	-0.079	1				
Probability	0.151	0.129	0.926	0.861	0.741	0.433					
NEG.EARN	-0.026	-0.047	-0.056	-0.001	-0.048	-0.073	-0.138	1			
Probability	0.799	0.643	0.580	0.995	0.635	0.474	0.170				
LEVERAGE	0.202**	0.165	0. 280***	0.555***	0.465***	-0.033	-0.059	0.319***	1		
Probability	0.044	0.101	0.005	0.000	0.000	0.743	0.558	0.001			
CAP.INTENSITY	-0.072	0.012	-0.031	-0.238**	-0.357***	0.067	-0.088	0.048	-0.489***	1	
Probability	0.474	0.906	0.758	0.017	0.000	0.508	0.383	0.638	0.000		
FDQ (C)_t-1_	0.473***	0.453***	0.408***	0.338***	0.386***	0.343***	-0.120	0.019	0.072	0.134	1
Probability	0.000	0.000	0.000	0.001	0.000	0.001	0.235	0.848	0.477	0.185	

*** is at 1%, ** is at 5%, *** and is at 10% significance. Probability refers to the statistical significance (alpha) value.

#### Unidimensional financial disclosure quality

Several studies have used accruals earnings quality as financial disclosure as a single dimension measure [36, 47, 49] whereas others have used a comprehensive multidimensional FDQ measure. This study used the decile ranked accruals earnings disclosure quality (AEDQ) as a robust measure. Table 7 shows that the results are consistent with the main model.

**Table 7:** Relationship between accrual earnings disclosure quality and sustainability disclosure quality.

	Hypothesis 1 (FDQ_t_)			Hypothesis 2 (FDQ_t+1_)		
Variable	Coef.	Std. error	Pr	Coef.	Std. error	Pr
SDQt	0.007**	0.003	0.014	0.009**	0.003	0.012
SIZE	0.935***	0.333	0.006	0.136	0.482	0.779
CFO	-0.001	0.001	0.354	-0.001	0.001	0.510
SALES	0.000	0.000	0.186	0.000	0.000	0.462
OP. CYCLE	-1.319***	0.399	0.001	0.031	0.529	0.953
NEG.EARNINGS	0.058	0.278	0.836	0.032	0.263	0.904
LEVERAGE	-0.040	0.032	0.219	0.012	0.046	0.798
CAP.INTENSITY	-1.764*	0.968	0.072	-1.057	1.342	0.433
FDQ_t-1_	0.280**	0.108	0.011			
FDQ_t_				0.403***	0.112	0.001
Constant	-4.258	3.598	0.24	0.294	4.684	0.950
R2	41.3%			41.9%		
F	0.000			0.000		
Observations	100			100		

*** and ** denote significance at the 1% and 5% levels, respectively.

The lagged AEDQ_t-1_ positively associates with AEDQ_t_ of the current period. SDQ_t_ is significantly associated with the financial disclosure quality of the current and future years. The model’s explanatory power has increased slightly from the current period (41.3 per cent) to the future period (41.9 per cent) AEDQ. There is no high correlation among variables. The mean-variance inflation factor (VIF) for regression on the Hypothesis 1 testing model is 1.86 (maximum 3.03 and minimum 1.06), and the mean VIF for regression on the Hypothesis 2 testing model is 1.94 (maximum 3.2 and minimum 1.14), indicating no multiple collinear relationships.

### Industry membership and state control as additional determinants of multidimensional financial disclosure quality

State intervention on environmental and sustainability disclosure has shown mixed results. For instance, EU directives have led to large companies making more disclosures [23]; a study with Bangladeshi Banks showed that regulatory pressures lead to authentic sustainability disclosure [24]; and a study conducted with firms in Peru showed that regulatory intervention on sustainability disclosure has no effect [25].

The Chinese milk scandal was a vital health safety concern, and firms with state ownership may have colluded with firms on SDQ (that is, regulatory capture theory) to be seen as trustworthy (organisational hypocrisy theory) by various stakeholders with façades to meet their information needs (organisational façade theory). This may have led to a slightly better explanation of the main models from SDQ_t_ and FDQ_t_ to SDQ_t_ and FDQ_t+1_. Industry membership can also influence this association. The study used the percentage of state ownership in shareholdings as proxy for state ownership. Firms classified as high profile were assigned 1 and those classified as low profile were assigned 0. High-profile industries included metals, banking and insurance, extractive, construction, telecommunication, electricity, transportation, oil and chemical, and food and beverage [44]. Results are reported in Table 8.

**Table 8:** Relationship between financial disclosure quality and sustainability disclosure quality (with industry membership and state ownership).

	Hypothesis 1 (FDQ_t_)			Hypothesis 2 (FDQ_t+1_)		
Variable	Coef.	Std. error	Pr	Coef.	Std. error	Pr
SDQt	0.026**	0.011	0.021	0.034**	0.014	0.014
SIZE	3.566**	1.373	0.011	0.253	1.909	0.895
CFO	-0.004	0.004	0.337	-0.003	0.005	0.468
SALES	-0.001	0.001	0.157	0.001	0.001	0.564
OP. CYCLE	-5.200	1.666***	0.002	0.159	1.936	0.935
NEG.EARNINGS	0.495	1.293	0.703	0.778	1.243	0.533
LEVERAGE	-0.155	0.179	0.389	-0.013	0.219	0.954
CAP.INTENSITY	-7.767	5.521	0.163	-7.188	6.881	0.299
FDQ_t-1_	0.282	0.110**	0.012			
FDQ_t_	-0.019	2.382	0.994	0.397***	0.113	0.001
Industry	2.706	3.270	0.41	1.387	2.523	0.584
State ownership	-16.308	14.670	0.269	3.066	3.195	0.34
Constant	0.026	0.011	0.021	3.150	18.426	0.865
R2	41.8%			42.9%		
F	0.000			0.000		
Observations	100			100		

*** and ** denote significance at the 1% and 5% levels, respectively.

There is no high correlation among variables. The VIF for regression on the extended Hypothesis 1 testing model is 2.0 (maximum 3.02 and minimum 1.06), and the mean VIF for regression on the expanded Hypothesis 2 testing model is 2.04 (maximum 3.3 and minimum 1.14), indicating no multiple collinear relationships.

Both state ownership and industry membership show no statistical significance in the expanded SDQ_t_ and FDQ_t_ model and SDQ_t_ and FDQ_t+1_ model. These results rule out the possibility of following year increasing explanation of the association between SDQ_t_ and FDQ_t+1_ using regulatory capture theory, organisational hypocrisy theory, and organisational façade theory. The FDQ_t_ has become a significant predictor of FDQ_t+1_. The findings of the expanded SDQ_t_ and FDQ_t+1_ model, showing SDQ_t_ and FDQ_t_ having statistical significance with FDQ_t+1_, support the increasing trustworthiness among all stakeholder groups and sample firms.

## Conclusion

This study finds that sustainability disclosure quality (SDQ) statistically associates with financial disclosure quality (FDQ). The SDQ in this study is stakeholder-relevant, objectively quantified economic, environmental, and social disclosure. This research posits a trustworthy relationship between socially reputable firms and their stakeholders, to demonstrate that current period SDQ is indicative of FDQ of the current and the future period, supporting the stakeholder theory in the Chinese context.

### Theoretical implications

Firms with a social reputation engage the trust of stakeholders, upholding the Confucian cultural values in China. A twist to the stakeholder theory in the Chinese context is not managing relationships to manage trustworthiness as a managerial activity [39], but building and maintaining relationships for the longer run, earning trustworthiness as a virtuous activity. The findings bring forth the vitality of Confucian virtue, Guanxi, understood through its direct connection with two other virtues: xinyong–trustworthiness, and renqing–mutual obligations. Research shows that other countries have required regulatory intervention to promote sustainability disclosure, under the presumption that societal cultural virtues are unable to promote such ideals of disclosure [22–25]. The phenomenon lends support to the conclusion of some researchers that Guanxi is unique to the Chinese culture [56, 57]. The contextual findings lead to a cultural stakeholder theory where underlying values of the societal culture are a condition facilitating organically mutual stakeholder relationships between firms and all the various stakeholders.

Future research can examine which dimension(s) of Hofstede culture simultaneously co-exist and promote Confucian-type virtues of trustworthiness and mutual obligations, as theoretical constructs. China comprises a unique set of Hofstede dimensions of culture scores [18]. A unique feature of China is its high long-term orientation score (87) where the society is modest, pragmatic, and thrifty; low indulgence score (24) where people restrain their freedom to gratify themselves based on their drives and emotions; and low uncertainty avoidance score (30) where people are less stressed and accommodating. At this stage, research provides little guidance on whether to associate these two virtues with a single or combined societal dimension. If long-term orientation is the strongest contributing dimension, then Japan (88) and Taiwan (93) would be fruitful research sites. If uncertainty avoidance is the strongest contributing dimension, then Vietnam (30) would be a useful research site [18].

### Managerial implications

The Chinese government can exercise regulatory powers over firms and the market. The government can design innovative schemes such as offering trustworthy firms base-point reductions in interest rates on borrowing or raising funds. Instrumentalising trustworthiness among firms can mitigate scandals such as the Sanlu milk contamination [58]. Although China has experienced rapid economic growth and has become a global economic leader, it has lagged in demonstrating trustworthiness due to various issues relating to security and secrecy [1]. It is a vital step for China to demonstrate the trustworthiness virtues of its culture to the rest of the world in order to claim a stake in global leadership, as global leaders become accepted due to their trustworthiness. Trustworthiness can help in boosting consumer confidence as well as foreign trade relations. Trustworthy firms can more readily receive resources and support [59].

Studies that use survey and content analysis techniques carry their own measurement biases. While this study took every possible care to minimise these, we acknowledge that we cannot entirely eliminate them. The sample size and generalising findings beyond socially reputable firms are limitations imposed by the sample selection. A future study can investigate with a sample selected not only across firm industry membership but also across nations, as social norms and ethical values of stakeholders contributing to stakeholder perceptions may differ among firm industry membership and countries. A future study can also investigate the relationship between FDQ and SDQ for specific stakeholder groups. Further, this study examined the relationship between FDQ and SDQ underpinned by the notion of trustworthiness, but future research can examine the relationship underpinned by the concept of altruism.

## Supporting information

S1 File (DOCX)
