# Supplementary material for: Financial disclosure quality and sustainability disclosure quality. A case in China
Source: PLoS One. 2021 May 28;16(5):e0250884. doi: 10.1371/journal.pone.0250884 (PMC8162600; doi:10.1371/journal.pone.0250884)
Supplement: S1 File — (DOCX) [file pone.0250884.s001.docx]

**Questionnaire**

***Economic (EC) Version***

**Part One**

**Instructions: Please indicate your response to each of the following disclosure types by circling the scale number that best describes your feeling.**

| ***Context Information for Understanding Corporate Performance*** | | |
| --- | --- | --- |
| ***Strategy and analysis*** | | |
| **Disclosure type** | **Typical example** | **Rating scale** |
| **1. Specific endeavour in non-quantitative terms** | “CSEC sticked to the goal of building an enterprise incorporating the Five-Model of “intrinsic safety, quality and efficiency, technological innovation, resource saving and harmonious development” and incorporated social responsibilities into the whole process of corporate strategic, cultural, production and operation activities” (Shenhua Energy, 2008, p.6). | **Unimportant Important**  **•┼――┼――┼――┼――┼――┼――┼――┼――┼――┼――┼―•**  **0 10 20 30 40 50 60 70 80 90 100** |
| ***Corporate profile*** |  |  |
| **Disclosure type** | **Typical example** | **Rating scale** |
| **1. General narrative information** | “Address: No. 55 Fuxingmennei Avenue, Xicheng District, Beijing, PRC” (ICBC, 2008, p.2). | **Unimportant Important**  **•┼――┼――┼――┼――┼――┼――┼――┼――┼――┼――┼―•**  **0 10 20 30 40 50 60 70 80 90 100** |
| **2. Specific endeavour in non-quantitative terms** | “The businesses of CSEC mainly cover production and sales of coal, railway and port transportation of coal-related material as well as the power generation and sales” (Shenhua Energy, 2008, preface). | **Unimportant Important**  **•┼――┼――┼――┼――┼――┼――┼――┼――┼――┼――┼―•**  **0 10 20 30 40 50 60 70 80 90 100** |
| **3. Quantified data** | “The Group has a total number of 138,368 employees” (China Mobile, 2008, p.5). | **Unimportant Important**  **•┼――┼――┼――┼――┼――┼――┼――┼――┼――┼――┼―•**  **0 10 20 30 40 50 60 70 80 90 100** |
| ***Report parameters*** |  |  |
| **Disclosure type** | **Typical example** | **Rating scale** |
| **1. General narrative information** | “The issues highlighted in the report are mainly related to our performances on the economic, environmental and social responsibilities fronts in 2008” (PetroChina, 2008, preface). | **Unimportant Important**  **•┼――┼――┼――┼――┼――┼――┼――┼――┼――┼――┼―•**  **0 10 20 30 40 50 60 70 80 90 100** |
| **2. Specific endeavour in non-quantitative terms** | “We are committed to observing and supporting the ten Principles advocated by the Global Compact in the fields of human rights, labor rights, environment protection and anti-corruption, using the ten Principles to guide our practices in fulfilling social responsibilities. Starting from this year, we will disclose our progress in keeping with the ten Principles in the Global Compact in our annual report. Please see the following table…” (PetroChina, 2008, p.48). | **Unimportant Important**  **•┼――┼――┼――┼――┼――┼――┼――┼――┼――┼――┼―•**  **0 10 20 30 40 50 60 70 80 90 100** |
| ***Governance, commitments and engagement*** | | |
| **Disclosure type** | **Typical example** | **Rating scale** |
| **1. General narrative information** | “The positions of chairman and president of the Bank are separate” (ICBC, 2008, p.22). | **Unimportant Important**  **•┼――┼――┼――┼――┼――┼――┼――┼――┼――┼――┼―•**  **0 10 20 30 40 50 60 70 80 90 100** |
| **2. Specific endeavour in non-quantitative terms** | “The Board of Directors has four board committees, namely the Audit Committee, the Investment and Development Committee, the Evaluation and Remuneration Committee, and the Health, Safety and Environment Committee. The Audit Committee is mainly responsible for…” (PetroChina, 2008, p.9). | **Unimportant Important**  **•┼――┼――┼――┼――┼――┼――┼――┼――┼――┼――┼―•**  **0 10 20 30 40 50 60 70 80 90 100** |
| **3. Quantified data** | “The Board of Directors is composed of 15 members, including the Chairman, 3 executive directors, 7 nonexecutive directors and 4 independent directors” (Bank of China, 2008, p.38). | **Unimportant Important**  **•┼――┼――┼――┼――┼――┼――┼――┼――┼――┼――┼―•**  **0 10 20 30 40 50 60 70 80 90 100** |
| ***Performance Information (Economic)*** | | |
| **Disclosure type** | **Typical example** | **Rating scale** |
| **1. General narrative information** | “The Company employs local residents first for selected post, a way to provide more jobs for local residents and to perform social responsibility for local economic development” (BaoSteel, 2008, p.23). | **Unimportant Important**  **•┼――┼――┼――┼――┼――┼――┼――┼――┼――┼――┼―•**  **0 10 20 30 40 50 60 70 80 90 100** |
| **2. Specific endeavour in non-quantitative terms** | “Cost cutting measures have been introduced, which focus on reducing administrative expenditures. The resources saved have been applied to managing crises, supporting key state projects and assisting customers” (Bank of China, 2008, p.25). | **Unimportant Important**  **•┼――┼――┼――┼――┼――┼――┼――┼――┼――┼――┼―•**  **0 10 20 30 40 50 60 70 80 90 100** |
| **3. Quantified performance data** | “As at 31 December 2008, the Company had received a total of State reward on technical reform on energy conservation of approximately RMB12.7 million” (Shenhua Energy, 2008, p.39). | **Unimportant Important**  **•┼――┼――┼――┼――┼――┼――┼――┼――┼――┼――┼―•**  **0 10 20 30 40 50 60 70 80 90 100** |
| **4. Quantified performance data relative to benchmarks** | “The Company’s taxation payments (billion yuan) are 30.1 in 2006, 42.1 in 2007 and 36.8 in 2008” (China Mobile, 2008, p.60). | **Unimportant Important**  **•┼――┼――┼――┼――┼――┼――┼――┼――┼――┼――┼―•**  **0 10 20 30 40 50 60 70 80 90 100** |
| **5. Quantified performance data at disaggregate level (e.g. plant, business unit, geographic segment)** | **“Within the huge investment of the West-East Gas Pipeline project, about RMB 34 billion went to the Western provinces, of which over RMB 20 billion went to Xinjiang, creating a huge consumption market and a large number of job opportunities. Meanwhile, the project has brought the economic structure adjustment of the East into a new level” (PetroChina, 2008, p.41).** | **Unimportant Important**  **•┼――┼――┼――┼――┼――┼――┼――┼――┼――┼――┼―•**  **0 10 20 30 40 50 60 70 80 90 100** |

***Please indicate any additional disclosure type that you feel should be included in the list and assign a weighting to it:***

| Disclosure type Typical example Unimportant Important  **•┼――┼――┼――┼――┼――┼――┼――┼――┼――┼――┼―•**  0 10 20 30 40 50 60 70 80 90 100 |
| --- |
|  |

**Part Two (Respondent’s profile)**

**Instructions: Please complete the following question. Your information will be kept strictly confidential.**

**What is your relationship with the firm that sends you this survey?**

Shareholder  Creditor  Government/Regulator  Employee

Customer  Supplier  Community  Media  Audit firm

Academic  Other (Please specify)

***Environmental (EN) Version***

**Part One**

**Instructions: Please indicate your response to each of the following disclosure types by circling the scale number that best describes your feeling.**

| ***Context Information for Understanding Corporate Performance*** | | |
| --- | --- | --- |
| ***Strategy and analysis*** | | |
| **Disclosure type** | **Typical example** | **Rating scale** |
| **1. Specific endeavour in non-quantitative terms** | “CSEC sticked to the goal of building an enterprise incorporating the Five-Model of “intrinsic safety, quality and efficiency, technological innovation, resource saving and harmonious development” and incorporated social responsibilities into the whole process of corporate strategic, cultural, production and operation activities” (Shenhua Energy, 2008, p.6). | **Unimportant Important**  **•┼――┼――┼――┼――┼――┼――┼――┼――┼――┼――┼―•**  **0 10 20 30 40 50 60 70 80 90 100** |
| ***Corporate profile*** |  |  |
| **Disclosure type** | **Typical example** | **Rating scale** |
| **1. General narrative information** | “Address: No. 55 Fuxingmennei Avenue, Xicheng District, Beijing, PRC” (ICBC, 2008, p.2). | **Unimportant Important**  **•┼――┼――┼――┼――┼――┼――┼――┼――┼――┼――┼―•**  **0 10 20 30 40 50 60 70 80 90 100** |
| **2. Specific endeavour in non-quantitative terms** | “The businesses of CSEC mainly cover production and sales of coal, railway and port transportation of coal-related material as well as the power generation and sales” (Shenhua Energy, 2008, preface). | **Unimportant Important**  **•┼――┼――┼――┼――┼――┼――┼――┼――┼――┼――┼―•**  **0 10 20 30 40 50 60 70 80 90 100** |
| **3. Quantified data** | “The Group has a total number of 138,368 employees” (China Mobile, 2008, p.5). | **Unimportant Important**  **•┼――┼――┼――┼――┼――┼――┼――┼――┼――┼――┼―•**  **0 10 20 30 40 50 60 70 80 90 100** |
| ***Report parameters*** |  |  |
| **Disclosure type** | **Typical example** | **Rating scale** |
| **1. General narrative information** | “The issues highlighted in the report are mainly related to our performances on the economic, environmental and social responsibilities fronts in 2008” (PetroChina, 2008, preface). | **Unimportant Important**  **•┼――┼――┼――┼――┼――┼――┼――┼――┼――┼――┼―•**  **0 10 20 30 40 50 60 70 80 90 100** |
| **2. Specific endeavour in non-quantitative terms** | “We are committed to observing and supporting the ten Principles advocated by the Global Compact in the fields of human rights, labor rights, environment protection and anti-corruption, using the ten Principles to guide our practices in fulfilling social responsibilities. Starting from this year, we will disclose our progress in keeping with the ten Principles in the Global Compact in our annual report. Please see the following table…” (PetroChina, 2008, p.48). | **Unimportant Important**  **•┼――┼――┼――┼――┼――┼――┼――┼――┼――┼――┼―•**  **0 10 20 30 40 50 60 70 80 90 100** |
| ***Governance, commitments and engagement*** | | |
| **Disclosure type** | **Typical example** | **Rating scale** |
| **1. General narrative information** | “The positions of chairman and president of the Bank are separate” (ICBC, 2008, p.22). | **Unimportant Important**  **•┼――┼――┼――┼――┼――┼――┼――┼――┼――┼――┼―•**  **0 10 20 30 40 50 60 70 80 90 100** |
| **2. Specific endeavour in non-quantitative terms** | “The Board of Directors has four board committees, namely the Audit Committee, the Investment and Development Committee, the Evaluation and Remuneration Committee, and the Health, Safety and Environment Committee. The Audit Committee is mainly responsible for…” (PetroChina, 2008, p.9). | **Unimportant Important**  **•┼――┼――┼――┼――┼――┼――┼――┼――┼――┼――┼―•**  **0 10 20 30 40 50 60 70 80 90 100** |
| **3. Quantified data** | “The Board of Directors is composed of 15 members, including the Chairman, 3 executive directors, 7 nonexecutive directors and 4 independent directors” (Bank of China, 2008, p.38). | **Unimportant Important**  **•┼――┼――┼――┼――┼――┼――┼――┼――┼――┼――┼―•**  **0 10 20 30 40 50 60 70 80 90 100** |
| ***Performance Information (Environmental)*** | | |
| **Disclosure type** | **Typical example** | **Rating scale** |
| **1. General narrative information** | “The company took energy conservation and emission reduction as important means to change the development modes” (PetroChina, 2008, p.30). | **Unimportant Important**  **•┼――┼――┼――┼――┼――┼――┼――┼――┼――┼――┼―•**  **0 10 20 30 40 50 60 70 80 90 100** |
| **2. Specific endeavour in non-quantitative terms** | “Baosteel focused on controlling the sulphur content of raw fuel and installing flue gas desulphurization facilities in the sintering factory and power plants for SO_2_ emission reduction” (BaoSteel, 2008, p.45). | **Unimportant Important**  **•┼――┼――┼――┼――┼――┼――┼――┼――┼――┼――┼―•**  **0 10 20 30 40 50 60 70 80 90 100** |
| **3. Quantified performance data** | “In terms of energy conservation and emission reduction, the company has set up an energy conservation and emission reduction fund, and the investment in energy conservation and emission reduction projects in 2008 amounted to a total of RMB1.39 billion” (Shenhua Energy, 2008, p.39). | **Unimportant Important**  **•┼――┼――┼――┼――┼――┼――┼――┼――┼――┼――┼―•**  **0 10 20 30 40 50 60 70 80 90 100** |
| **4. Quantified performance data relative to benchmarks** | “Our total Carbon Dioxide emissions (million tonnes) are 5.4 in 2006, 6.9 in 2007 and 7.9 in 2008” (China Mobile, 2008, p.40). | **Unimportant Important**  **•┼――┼――┼――┼――┼――┼――┼――┼――┼――┼――┼―•**  **0 10 20 30 40 50 60 70 80 90 100** |
| **5. Quantified performance data at disaggregate level (e.g. plant, business unit, geographic segment)** | **“In Chengdu branch, energy consumption was reduced and operating costs were saved by strengthening micro-management. For example, standardized control was applied to the on/off time of central air-conditioning while allowing timely notices to be made to the property management for adjustments based on the temperature of the day. In Beijing branch, the lighting source for the front access light box was changed from ordinary fluorescent tubes to energy saving tubes, saving approximately 30% power consumption” (Merchants Bank, 2008, p.21).** | **Unimportant Important**  **•┼――┼――┼――┼――┼――┼――┼――┼――┼――┼――┼―•**  **0 10 20 30 40 50 60 70 80 90 100** |

***Please indicate any additional disclosure type that you feel should be included in the list and assign a weighting to it:***

| Disclosure type Typical example Unimportant Important  **•┼――┼――┼――┼――┼――┼――┼――┼――┼――┼――┼―•**  0 10 20 30 40 50 60 70 80 90 100 |
| --- |
|  |

**Part Two (Respondent’s profile)**

**Instructions: Please complete the following question. Your information will be kept strictly confidential.**

**What is your relationship with the firm that sends you this survey?**

Shareholder  Creditor  Government/Regulator  Employee

Customer  Supplier  Community  Media  Audit firm

Academic  Other (Please specify)

***Labour Practices (LA) Version***

**Part One**

**Instructions: Please indicate your response to each of the following disclosure types by circling the scale number that best describes your feeling.**

| ***Context Information for Understanding Corporate Performance*** | | |
| --- | --- | --- |
| ***Strategy and analysis*** | | |
| **Disclosure type** | **Typical example** | **Rating scale** |
| **1. Specific endeavour in non-quantitative terms** | “CSEC sticked to the goal of building an enterprise incorporating the Five-Model of “intrinsic safety, quality and efficiency, technological innovation, resource saving and harmonious development” and incorporated social responsibilities into the whole process of corporate strategic, cultural, production and operation activities” (Shenhua Energy, 2008, p.6). | **Unimportant Important**  **•┼――┼――┼――┼――┼――┼――┼――┼――┼――┼――┼―•**  **0 10 20 30 40 50 60 70 80 90 100** |
| ***Corporate profile*** |  |  |
| **Disclosure type** | **Typical example** | **Rating scale** |
| **1. General narrative information** | “Address: No. 55 Fuxingmennei Avenue, Xicheng District, Beijing, PRC” (ICBC, 2008, p.2). | **Unimportant Important**  **•┼――┼――┼――┼――┼――┼――┼――┼――┼――┼――┼―•**  **0 10 20 30 40 50 60 70 80 90 100** |
| **2. Specific endeavour in non-quantitative terms** | “The businesses of CSEC mainly cover production and sales of coal, railway and port transportation of coal-related material as well as the power generation and sales” (Shenhua Energy, 2008, preface). | **Unimportant Important**  **•┼――┼――┼――┼――┼――┼――┼――┼――┼――┼――┼―•**  **0 10 20 30 40 50 60 70 80 90 100** |
| **3. Quantified data** | “The Group has a total number of 138,368 employees” (China Mobile, 2008, p.5). | **Unimportant Important**  **•┼――┼――┼――┼――┼――┼――┼――┼――┼――┼――┼―•**  **0 10 20 30 40 50 60 70 80 90 100** |
| ***Report parameters*** |  |  |
| **Disclosure type** | **Typical example** | **Rating scale** |
| **1. General narrative information** | “The issues highlighted in the report are mainly related to our performances on the economic, environmental and social responsibilities fronts in 2008” (PetroChina, 2008, preface). | **Unimportant Important**  **•┼――┼――┼――┼――┼――┼――┼――┼――┼――┼――┼―•**  **0 10 20 30 40 50 60 70 80 90 100** |
| **2. Specific endeavour in non-quantitative terms** | “We are committed to observing and supporting the ten Principles advocated by the Global Compact in the fields of human rights, labor rights, environment protection and anti-corruption, using the ten Principles to guide our practices in fulfilling social responsibilities. Starting from this year, we will disclose our progress in keeping with the ten Principles in the Global Compact in our annual report. Please see the following table…” (PetroChina, 2008, p.48). | **Unimportant Important**  **•┼――┼――┼――┼――┼――┼――┼――┼――┼――┼――┼―•**  **0 10 20 30 40 50 60 70 80 90 100** |
| ***Governance, commitments and engagement*** | | |
| **Disclosure type** | **Typical example** | **Rating scale** |
| **1. General narrative information** | “The positions of chairman and president of the Bank are separate” (ICBC, 2008, p.22). | **Unimportant Important**  **•┼――┼――┼――┼――┼――┼――┼――┼――┼――┼――┼―•**  **0 10 20 30 40 50 60 70 80 90 100** |
| **2. Specific endeavour in non-quantitative terms** | “The Board of Directors has four board committees, namely the Audit Committee, the Investment and Development Committee, the Evaluation and Remuneration Committee, and the Health, Safety and Environment Committee. The Audit Committee is mainly responsible for…” (PetroChina, 2008, p.9). | **Unimportant Important**  **•┼――┼――┼――┼――┼――┼――┼――┼――┼――┼――┼―•**  **0 10 20 30 40 50 60 70 80 90 100** |
| **3. Quantified data** | “The Board of Directors is composed of 15 members, including the Chairman, 3 executive directors, 7 nonexecutive directors and 4 independent directors” (Bank of China, 2008, p.38). | **Unimportant Important**  **•┼――┼――┼――┼――┼――┼――┼――┼――┼――┼――┼―•**  **0 10 20 30 40 50 60 70 80 90 100** |
| ***Performance Information (Labour Practices)*** | | |
| **Disclosure type** | **Typical example** | **Rating scale** |
| **1. General narrative information** | “With respect to employee health and safety, we strictly implement national laws and regulations related to labour protection and safety production” (China Mobile, 2008, p.19). | **Unimportant Important**  **•┼――┼――┼――┼――┼――┼――┼――┼――┼――┼――┼―•**  **0 10 20 30 40 50 60 70 80 90 100** |
| **2. Specific endeavour in non-quantitative terms** | “BOC provides employees with benefits that include social security, a housing provident fund, statutory holidays, enterprise annuity, and supplementary medical insurance” (Bank of China, 2008, p.47). | **Unimportant Important**  **•┼――┼――┼――┼――┼――┼――┼――┼――┼――┼――┼―•**  **0 10 20 30 40 50 60 70 80 90 100** |
| **3. Quantified performance data** | “The capital investment in prevention of occupational diseases was approximately 78 million in 2008” (Shenhua Energy, 2008, p.66). | **Unimportant Important**  **•┼――┼――┼――┼――┼――┼――┼――┼――┼――┼――┼―•**  **0 10 20 30 40 50 60 70 80 90 100** |
| **4. Quantified performance data relative to benchmarks** | “The number of on-the-job training employees increases year by year, 5164 in 2006, 6232 in 2007 and 7657 in 2008” (Bank of China, 2008, p.48). | **Unimportant Important**  **•┼――┼――┼――┼――┼――┼――┼――┼――┼――┼――┼―•**  **0 10 20 30 40 50 60 70 80 90 100** |
| **5. Quantified performance data at disaggregate level (e.g. plant, business unit, geographic segment)** | **“As at the end of 2008, the Bank had 385,609 employees, an increase of 3,896 persons compared with the end of prior year, of whom 221 are employees in major domestic holding companies and 2,697 are local employees in overseas institutions. Among the employees in domestic institutions, 39,124 are engaged in the corporate banking segment, 149,166 in personal banking segment, 4,522 in treasury operations segment, 87,040 in financial and accounting matters, and 103,060 in other specializations” (ICBC, 2008, p.80).** | **Unimportant Important**  **•┼――┼――┼――┼――┼――┼――┼――┼――┼――┼――┼―•**  **0 10 20 30 40 50 60 70 80 90 100** |

***Please indicate any additional disclosure type that you feel should be included in the list and assign a weighting to it:***

| Disclosure type Typical example Unimportant Important  **•┼――┼――┼――┼――┼――┼――┼――┼――┼――┼――┼―•**  0 10 20 30 40 50 60 70 80 90 100 |
| --- |
|  |

**Part Two (Respondent’s profile)**

**Instructions: Please complete the following question. Your information will be kept strictly confidential.**

**What is your relationship with the firm that sends you this survey?**

Shareholder  Creditor  Government/Regulator  Employee

Customer  Supplier  Community  Media  Audit firm

Academic  Other (Please specify)

***Human Rights (HR) Version***

**Part One**

**Instructions: Please indicate your response to each of the following disclosure types by circling the scale number that best describes your feeling.**

| ***Context Information for Understanding Corporate Performance*** | | |
| --- | --- | --- |
| ***Strategy and analysis*** | | |
| **Disclosure type** | **Typical example** | **Rating scale** |
| **1. Specific endeavour in non-quantitative terms** | “CSEC sticked to the goal of building an enterprise incorporating the Five-Model of “intrinsic safety, quality and efficiency, technological innovation, resource saving and harmonious development” and incorporated social responsibilities into the whole process of corporate strategic, cultural, production and operation activities” (Shenhua Energy, 2008, p.6). | **Unimportant Important**  **•┼――┼――┼――┼――┼――┼――┼――┼――┼――┼――┼―•**  **0 10 20 30 40 50 60 70 80 90 100** |
| ***Corporate profile*** |  |  |
| **Disclosure type** | **Typical example** | **Rating scale** |
| **1. General narrative information** | “Address: No. 55 Fuxingmennei Avenue, Xicheng District, Beijing, PRC” (ICBC, 2008, p.2). | **Unimportant Important**  **•┼――┼――┼――┼――┼――┼――┼――┼――┼――┼――┼―•**  **0 10 20 30 40 50 60 70 80 90 100** |
| **2. Specific endeavour in non-quantitative terms** | “The businesses of CSEC mainly cover production and sales of coal, railway and port transportation of coal-related material as well as the power generation and sales” (Shenhua Energy, 2008, preface). | **Unimportant Important**  **•┼――┼――┼――┼――┼――┼――┼――┼――┼――┼――┼―•**  **0 10 20 30 40 50 60 70 80 90 100** |
| **3. Quantified data** | “The Group has a total number of 138,368 employees” (China Mobile, 2008, p.5). | **Unimportant Important**  **•┼――┼――┼――┼――┼――┼――┼――┼――┼――┼――┼―•**  **0 10 20 30 40 50 60 70 80 90 100** |
| ***Report parameters*** |  |  |
| **Disclosure type** | **Typical example** | **Rating scale** |
| **1. General narrative information** | “The issues highlighted in the report are mainly related to our performances on the economic, environmental and social responsibilities fronts in 2008” (PetroChina, 2008, preface). | **Unimportant Important**  **•┼――┼――┼――┼――┼――┼――┼――┼――┼――┼――┼―•**  **0 10 20 30 40 50 60 70 80 90 100** |
| **2. Specific endeavour in non-quantitative terms** | “We are committed to observing and supporting the ten Principles advocated by the Global Compact in the fields of human rights, labor rights, environment protection and anti-corruption, using the ten Principles to guide our practices in fulfilling social responsibilities. Starting from this year, we will disclose our progress in keeping with the ten Principles in the Global Compact in our annual report. Please see the following table…” (PetroChina, 2008, p.48). | **Unimportant Important**  **•┼――┼――┼――┼――┼――┼――┼――┼――┼――┼――┼―•**  **0 10 20 30 40 50 60 70 80 90 100** |
| ***Governance, commitments and engagement*** | | |
| **Disclosure type** | **Typical example** | **Rating scale** |
| **1. General narrative information** | “The positions of chairman and president of the Bank are separate” (ICBC, 2008, p.22). | **Unimportant Important**  **•┼――┼――┼――┼――┼――┼――┼――┼――┼――┼――┼―•**  **0 10 20 30 40 50 60 70 80 90 100** |
| **2. Specific endeavour in non-quantitative terms** | “The Board of Directors has four board committees, namely the Audit Committee, the Investment and Development Committee, the Evaluation and Remuneration Committee, and the Health, Safety and Environment Committee. The Audit Committee is mainly responsible for…” (PetroChina, 2008, p.9). | **Unimportant Important**  **•┼――┼――┼――┼――┼――┼――┼――┼――┼――┼――┼―•**  **0 10 20 30 40 50 60 70 80 90 100** |
| **3. Quantified data** | “The Board of Directors is composed of 15 members, including the Chairman, 3 executive directors, 7 nonexecutive directors and 4 independent directors” (Bank of China, 2008, p.38). | **Unimportant Important**  **•┼――┼――┼――┼――┼――┼――┼――┼――┼――┼――┼―•**  **0 10 20 30 40 50 60 70 80 90 100** |
| ***Performance Information (Human Rights)*** | | |
| **Disclosure type** | **Typical example** | **Rating scale** |
| **1. General narrative information** | “We are committed to the principles of equal pay for equal work and gender and racial equality” (China Mobile, 2008, p.19). | **Unimportant Important**  **•┼――┼――┼――┼――┼――┼――┼――┼――┼――┼――┼―•**  **0 10 20 30 40 50 60 70 80 90 100** |
| **2. Specific endeavour in non-quantitative terms** | “The Company pays due attention to employees from ethnic minorities. Minority allowances are paid and Moslem restaurants are provided for these employees. Attention has been paid to appoint employees from ethnic minorities to some important management posts of the Company” (BaoSteel, 2008, p.24). | **Unimportant Important**  **•┼――┼――┼――┼――┼――┼――┼――┼――┼――┼――┼―•**  **0 10 20 30 40 50 60 70 80 90 100** |
| **3. Quantified performance data** | “The system of ‘4 shifts with 6 hours for each shift’ is implemented in power plants and certain coal mines, which helped to substantially ease the labour intensity of front-line staff” (Shenhua Energy, 2008, p.33). | **Unimportant Important**  **•┼――┼――┼――┼――┼――┼――┼――┼――┼――┼――┼―•**  **0 10 20 30 40 50 60 70 80 90 100** |
| **4. Quantified performance data relative to benchmarks** | “The second session of our Staff Representative Assembly was held in November 2008. Over 360 staff representatives and nearly 60 non-voting representatives attended the meeting, the number of representatives being higher than that of last session” (Construction Bank, 2008, p.112). | **Unimportant Important**  **•┼――┼――┼――┼――┼――┼――┼――┼――┼――┼――┼―•**  **0 10 20 30 40 50 60 70 80 90 100** |
| **5. Quantified performance data at disaggregate level (e.g. plant, business unit, geographic segment)** | **“During the reporting period, the Bank held 4,089 employees' representative meetings in total with 40,430 proposals from the employees' representatives, and of which 32,961 (of which 824 from Beijing branch and 798 from Shanghai branch) were fulfilled at the rate of 81.5%” (ICBC, 2008, p.82).** | **Unimportant Important**  **•┼――┼――┼――┼――┼――┼――┼――┼――┼――┼――┼―•**  **0 10 20 30 40 50 60 70 80 90 100** |

***Please indicate any additional disclosure type that you feel should be included in the list and assign a weighting to it:***

| Disclosure type Typical example Unimportant Important  **•┼――┼――┼――┼――┼――┼――┼――┼――┼――┼――┼―•**  0 10 20 30 40 50 60 70 80 90 100 |
| --- |
|  |

**Part Two (Respondent’s profile)**

**Instructions: Please complete the following question. Your information will be kept strictly confidential.**

**What is your relationship with the firm that sends you this survey?**

Shareholder  Creditor  Government/Regulator  Employee

Customer  Supplier  Community  Media  Audit firm

Academic  Other (Please specify)

***Society (SO) Version***

**Part One**

**Instructions: Please indicate your response to each of the following disclosure types by circling the scale number that best describes your feeling.**

| ***Context Information for Understanding Corporate Performance*** | | |
| --- | --- | --- |
| ***Strategy and analysis*** | | |
| **Disclosure type** | **Typical example** | **Rating scale** |
| **1. Specific endeavour in non-quantitative terms** | “CSEC sticked to the goal of building an enterprise incorporating the Five-Model of “intrinsic safety, quality and efficiency, technological innovation, resource saving and harmonious development” and incorporated social responsibilities into the whole process of corporate strategic, cultural, production and operation activities” (Shenhua Energy, 2008, p.6). | **Unimportant Important**  **•┼――┼――┼――┼――┼――┼――┼――┼――┼――┼――┼―•**  **0 10 20 30 40 50 60 70 80 90 100** |
| ***Corporate profile*** |  |  |
| **Disclosure type** | **Typical example** | **Rating scale** |
| **1. General narrative information** | “Address: No. 55 Fuxingmennei Avenue, Xicheng District, Beijing, PRC” (ICBC, 2008, p.2). | **Unimportant Important**  **•┼――┼――┼――┼――┼――┼――┼――┼――┼――┼――┼―•**  **0 10 20 30 40 50 60 70 80 90 100** |
| **2. Specific endeavour in non-quantitative terms** | “The businesses of CSEC mainly cover production and sales of coal, railway and port transportation of coal-related material as well as the power generation and sales” (Shenhua Energy, 2008, preface). | **Unimportant Important**  **•┼――┼――┼――┼――┼――┼――┼――┼――┼――┼――┼―•**  **0 10 20 30 40 50 60 70 80 90 100** |
| **3. Quantified data** | “The Group has a total number of 138,368 employees” (China Mobile, 2008, p.5). | **Unimportant Important**  **•┼――┼――┼――┼――┼――┼――┼――┼――┼――┼――┼―•**  **0 10 20 30 40 50 60 70 80 90 100** |
| ***Report parameters*** |  |  |
| **Disclosure type** | **Typical example** | **Rating scale** |
| **1. General narrative information** | “The issues highlighted in the report are mainly related to our performances on the economic, environmental and social responsibilities fronts in 2008” (PetroChina, 2008, preface). | **Unimportant Important**  **•┼――┼――┼――┼――┼――┼――┼――┼――┼――┼――┼―•**  **0 10 20 30 40 50 60 70 80 90 100** |
| **2. Specific endeavour in non-quantitative terms** | “We are committed to observing and supporting the ten Principles advocated by the Global Compact in the fields of human rights, labor rights, environment protection and anti-corruption, using the ten Principles to guide our practices in fulfilling social responsibilities. Starting from this year, we will disclose our progress in keeping with the ten Principles in the Global Compact in our annual report. Please see the following table…” (PetroChina, 2008, p.48). | **Unimportant Important**  **•┼――┼――┼――┼――┼――┼――┼――┼――┼――┼――┼―•**  **0 10 20 30 40 50 60 70 80 90 100** |
| ***Governance, commitments and engagement*** | | |
| **Disclosure type** | **Typical example** | **Rating scale** |
| **1. General narrative information** | “The positions of chairman and president of the Bank are separate” (ICBC, 2008, p.22). | **Unimportant Important**  **•┼――┼――┼――┼――┼――┼――┼――┼――┼――┼――┼―•**  **0 10 20 30 40 50 60 70 80 90 100** |
| **2. Specific endeavour in non-quantitative terms** | “The Board of Directors has four board committees, namely the Audit Committee, the Investment and Development Committee, the Evaluation and Remuneration Committee, and the Health, Safety and Environment Committee. The Audit Committee is mainly responsible for…” (PetroChina, 2008, p.9). | **Unimportant Important**  **•┼――┼――┼――┼――┼――┼――┼――┼――┼――┼――┼―•**  **0 10 20 30 40 50 60 70 80 90 100** |
| **3. Quantified data** | “The Board of Directors is composed of 15 members, including the Chairman, 3 executive directors, 7 nonexecutive directors and 4 independent directors” (Bank of China, 2008, p.38). | **Unimportant Important**  **•┼――┼――┼――┼――┼――┼――┼――┼――┼――┼――┼―•**  **0 10 20 30 40 50 60 70 80 90 100** |
| ***Performance Information (Society)*** | | |
| **Disclosure type** | Typical example | **Rating scale** |
| **1. General narrative information** | “The Company strengthens anti-corruption education to improve the ability to fight against corruption” (Shenhua Energy, 2008, p.24). | **Unimportant Important**  **•┼――┼――┼――┼――┼――┼――┼――┼――┼――┼――┼―•**  **0 10 20 30 40 50 60 70 80 90 100** |
| **2. Specific endeavour in non-quantitative terms** | “In 2008, we continued to implement the Rural Program and meet the commitment to rural development. By extending the reach of our ‘three networks’, we benefited the rural residents, rural businesses and rural governments and supported the development of Chinese rural areas” (China Mobile, 2008, p.22). | **Unimportant Important**  **•┼――┼――┼――┼――┼――┼――┼――┼――┼――┼――┼―•**  **0 10 20 30 40 50 60 70 80 90 100** |
| **3. Quantified performance data** | “Each year, the Company spends more than RMB 150 billion on purchasing materials, thus directly promoting the industries of steel, construction materials, machinery, and electronics” (PetroChina, 2008, p.41). | **Unimportant Important**  **•┼――┼――┼――┼――┼――┼――┼――┼――┼――┼――┼―•**  **0 10 20 30 40 50 60 70 80 90 100** |
| **4. Quantified performance data relative to benchmarks** | “The education donation (RMB10K) increases year by year, with 1,645 in 2006, 4,549 in 2007 and 12,968 in 2008” (PetroChina, 2008, p.49). | **Unimportant Important**  **•┼――┼――┼――┼――┼――┼――┼――┼――┼――┼――┼―•**  **0 10 20 30 40 50 60 70 80 90 100** |
| **5. Quantified performance data at disaggregate level (e.g. plant, business unit, geographic segment)** | **“After the quake, all the overseas institutions of the bank supported the affected population by various means. ICBC Indonesia opened a free-charge donation remittance channel to the whole country, and transmitted more than USD500,000 of donation to the Ministry of Civil Affairs, the Red Cross Society of China and the China Charity Federation; New York Branch donated to the 150 undergraduates in State University of New York at Stony Brook, who came from Sichuan under the ‘China 150 Program’” (ICBC, 2008, p.35).** | **Unimportant Important**  **•┼――┼――┼――┼――┼――┼――┼――┼――┼――┼――┼―•**  **0 10 20 30 40 50 60 70 80 90 100** |

***Please indicate any additional disclosure type that you feel should be included in the list and assign a weighting to it:***

| Disclosure type Typical example Unimportant Important  **•┼――┼――┼――┼――┼――┼――┼――┼――┼――┼――┼―•**  0 10 20 30 40 50 60 70 80 90 100 |
| --- |
|  |

**Part Two (Respondent’s profile)**

**Instructions: Please complete the following question. Your information will be kept strictly confidential.**

**What is your relationship with the firm that sends you this survey?**

Shareholder  Creditor  Government/Regulator  Employee

Customer  Supplier  Community  Media  Audit firm

Academic  Other (Please specify)

***Product Responsibility (PR) Version***

**Part One**

**Instructions: Please indicate your response to each of the following disclosure types by circling the scale number that best describes your feeling.**

| ***Context Information for Understanding Corporate Performance*** | | |
| --- | --- | --- |
| ***Strategy and analysis*** | | |
| **Disclosure type** | **Typical example** | **Rating scale** |
| **1. Specific endeavour in non-quantitative terms** | “CSEC sticked to the goal of building an enterprise incorporating the Five-Model of “intrinsic safety, quality and efficiency, technological innovation, resource saving and harmonious development” and incorporated social responsibilities into the whole process of corporate strategic, cultural, production and operation activities” (Shenhua Energy, 2008, p.6). | **Unimportant Important**  **•┼――┼――┼――┼――┼――┼――┼――┼――┼――┼――┼―•**  **0 10 20 30 40 50 60 70 80 90 100** |
| ***Corporate profile*** |  |  |
| **Disclosure type** | **Typical example** | **Rating scale** |
| **1. General narrative information** | “Address: No. 55 Fuxingmennei Avenue, Xicheng District, Beijing, PRC” (ICBC, 2008, p.2). | **Unimportant Important**  **•┼――┼――┼――┼――┼――┼――┼――┼――┼――┼――┼―•**  **0 10 20 30 40 50 60 70 80 90 100** |
| **2. Specific endeavour in non-quantitative terms** | “The businesses of CSEC mainly cover production and sales of coal, railway and port transportation of coal-related material as well as the power generation and sales” (Shenhua Energy, 2008, preface). | **Unimportant Important**  **•┼――┼――┼――┼――┼――┼――┼――┼――┼――┼――┼―•**  **0 10 20 30 40 50 60 70 80 90 100** |
| **3. Quantified data** | “The Group has a total number of 138,368 employees” (China Mobile, 2008, p.5). | **Unimportant Important**  **•┼――┼――┼――┼――┼――┼――┼――┼――┼――┼――┼―•**  **0 10 20 30 40 50 60 70 80 90 100** |
| ***Report parameters*** |  |  |
| **Disclosure type** | **Typical example** | **Rating scale** |
| **1. General narrative information** | “The issues highlighted in the report are mainly related to our performances on the economic, environmental and social responsibilities fronts in 2008” (PetroChina, 2008, preface). | **Unimportant Important**  **•┼――┼――┼――┼――┼――┼――┼――┼――┼――┼――┼―•**  **0 10 20 30 40 50 60 70 80 90 100** |
| **2. Specific endeavour in non-quantitative terms** | “We are committed to observing and supporting the ten Principles advocated by the Global Compact in the fields of human rights, labor rights, environment protection and anti-corruption, using the ten Principles to guide our practices in fulfilling social responsibilities. Starting from this year, we will disclose our progress in keeping with the ten Principles in the Global Compact in our annual report. Please see the following table…” (PetroChina, 2008, p.48). | **Unimportant Important**  **•┼――┼――┼――┼――┼――┼――┼――┼――┼――┼――┼―•**  **0 10 20 30 40 50 60 70 80 90 100** |
| ***Governance, commitments and engagement*** | | |
| **Disclosure type** | **Typical example** | **Rating scale** |
| **1. General narrative information** | “The positions of chairman and president of the Bank are separate” (ICBC, 2008, p.22). | **Unimportant Important**  **•┼――┼――┼――┼――┼――┼――┼――┼――┼――┼――┼―•**  **0 10 20 30 40 50 60 70 80 90 100** |
| **2. Specific endeavour in non-quantitative terms** | “The Board of Directors has four board committees, namely the Audit Committee, the Investment and Development Committee, the Evaluation and Remuneration Committee, and the Health, Safety and Environment Committee. The Audit Committee is mainly responsible for…” (PetroChina, 2008, p.9). | **Unimportant Important**  **•┼――┼――┼――┼――┼――┼――┼――┼――┼――┼――┼―•**  **0 10 20 30 40 50 60 70 80 90 100** |
| **3. Quantified data** | “The Board of Directors is composed of 15 members, including the Chairman, 3 executive directors, 7 nonexecutive directors and 4 independent directors” (Bank of China, 2008, p.38). | **Unimportant Important**  **•┼――┼――┼――┼――┼――┼――┼――┼――┼――┼――┼―•**  **0 10 20 30 40 50 60 70 80 90 100** |
| ***Performance Information (Product Responsibility)*** | | |
| **Disclosure type** | **Typical example** | **Rating scale** |
| **1. General narrative information** | “The company signed confidentiality agreements with employees from the sales department to keep customer privacy” (BaoSteel, 2008, p.29). | **Unimportant Important**  **•┼――┼――┼――┼――┼――┼――┼――┼――┼――┼――┼―•**  **0 10 20 30 40 50 60 70 80 90 100** |
| **2. Specific endeavour in non-quantitative terms** | “The key points of the Company’s customer relations included dedication in fulfilling contracts, provision of coal quality assurance for customers, improvement in the after-sales service system and customized product development based on customers’ needs” (Shenhua Energy, 2008, p.14). | **Unimportant Important**  **•┼――┼――┼――┼――┼――┼――┼――┼――┼――┼――┼―•**  **0 10 20 30 40 50 60 70 80 90 100** |
| **3. Quantified performance data** | “In 2008, clients’ satisfaction score was above 90” (BaoSteel, 2008, p.30). | **Unimportant Important**  **•┼――┼――┼――┼――┼――┼――┼――┼――┼――┼――┼―•**  **0 10 20 30 40 50 60 70 80 90 100** |
| **4. Quantified performance data relative to benchmarks** | “In 2008, our overall customer satisfaction scores increased to 81.3, compared with 80.8 in 2007 and 79.6 in 2006” (China Mobile, 2008, p.17). | **Unimportant Important**  **•┼――┼――┼――┼――┼――┼――┼――┼――┼――┼――┼―•**  **0 10 20 30 40 50 60 70 80 90 100** |
| **5. Quantified performance data at disaggregate level (e.g. plant, business unit, geographic segment)** | **“Satisfaction investigation was made to 1,800 corporate customers and 3,600 personal customers by preparing and issuing the customer satisfaction questionnaires. According to the investigation, the corporate and personal customer satisfaction rates reached 86.44% and 85.88% respectively” (ICBC, 2008, p.94).** | **Unimportant Important**  **•┼――┼――┼――┼――┼――┼――┼――┼――┼――┼――┼―•**  **0 10 20 30 40 50 60 70 80 90 100** |

***Please indicate any additional disclosure type that you feel should be included in the list and assign a weighting to it:***

| Disclosure type Typical example Unimportant Important  **•┼――┼――┼――┼――┼――┼――┼――┼――┼――┼――┼―•**  0 10 20 30 40 50 60 70 80 90 100 |
| --- |
|  |

**Part Two (Respondent’s profile)**

**Instructions: Please complete the following question. Your information will be kept strictly confidential.**

**What is your relationship with the firm that sends you this survey?**

Shareholder  Creditor  Government/Regulator  Employee

Customer  Supplier  Community  Media  Audit firm

Academic  Other (Please specify)
